# Novel Reassortant H5N2 Highly Pathogenic Avian Influenza Viruses from Backyard Poultry in Mexico

**DOI:** 10.3390/v18030337

**Published:** 2026-03-10

**Authors:** Mario Solís-Hernández, Guillermo Orta-Pineda, Carlos Javier Alcazar-Ramiro, Montserrat Amaranta Velázquez-Vázquez, Claudia Garnica-Rivera, Marisol Karina Rocha-Martínez, Nadia Carrillo-Guzmán, Ignacio Eliseo Tetla-Zapotitla, Israel Tiburcio-Sánchez, Héctor Javier Piña-Trevilla, Francisco José Liljehult-Fuentes, Armando García-López

**Affiliations:** 1 Comisión México-Estados Unidos para la Prevención de la Fiebre Aftosa y otras Enfermedades Exóticas de los Animales (CPA), SENASICA, Mexico City 05110, Mexico; 2 Centro Nacional de Referencia en Parasitología Animal y Tecnología Analítica (CENAPA), SENASICA, Jiutepec 62574, Mexico; marisol.rocha@senasica.gob.mx (M.K.R.-M.);

**Keywords:** avian influenza virus, backyard poultry systems, genomic reassortment, epidemiological surveillance, H5N2 reassortants

## Abstract

Highly pathogenic influenza A viruses of the H5 subtype continue to diversify worldwide through mutation and genetic reassortment, generating novel variants with unpredictable consequences under the One Health approach. Between 2024 and 2025, five outbreaks of avian influenza A viruses were detected in backyard poultry across Michoacán, Estado de México, and Ciudad de México. We conducted molecular and genetic characterization of five highly pathogenic H5N2 viruses isolated from these events. All cases tested positive for influenza A virus and the H5 hemagglutinin, exhibiting high pathogenicity with intravenous pathogenicity index values ranging from 2.88 to 3.0. Whole-genome sequencing revealed novel reassortants containing hemagglutinin from Eurasian H5N1 clade 2.3.4.4b and neuraminidase from the endemic Mexican H5N2 lineage. The viral genome of the isolate from Michoacán contained six segments derived from Eurasian H5N1 viruses introduced into North America in 2021–2022, while nucleoprotein and neuraminidase originated from Mexican H5N2 viruses. In contrast, viruses from Estado de México and Ciudad de México contained five H5N1-derived segments and incorporated polymerase basic protein 1, nucleoprotein, and neuraminidase from low-pathogenic H5N2 viruses circulating in 2024. Phylogenetic analyses confirmed the emergence of a distinct H5N2 Mexican sublineage, providing evidence of active viral reassortment and local evolutionary processes in Mexico.

## 1. Introduction

Influenza A viruses (IAV) represent a significant concern for global health, not only because of their pathogenicity and mortality rates in birds, but also due to their extraordinary adaptive and evolutionary capacity, which allows them to infect other host species. The emergence and spread of highly pathogenic avian influenza (HPAI) viruses, particularly the H5 subtype, have profound implications for food security, global poultry trade, animal and public health, emphasizing their relevance within the One Health approach [1]. These viruses can rapidly undergo genetic mutation and reassortment, facilitating adaptation to new environments and host species, including mammals [2]. Since the first detection of the highly pathogenic influenza A H5 virus in 1996, through its recent spillover events across Europe, Africa, the Americas, and Antarctica, these viruses have drawn global attention due to their capacity to infect avian populations, several mammalian species, and humans [3].

H5 influenza viruses have been classified into multiple clades and subclades through phylogenetic analyses of the hemagglutinin (HA) gene. The introduction of clade 2.3.4.4b H5N1 into North America in 2021, derived from the A/Goose/Guangdong/1/1996 lineage (GsGd), signaled the emergence of a new highly pathogenic subtype [4]. Overlapping poultry populations with the wildlife birds facilitated lineage coexistence and genetic reassortment, generating diverse H5Nx variants with novel genomic constellations [5,6,7]. This continuous diversification has produced multiple lineages with distinct pathogenicity and pandemic potential, host ranges, and increased geographic distributions, thereby complicating surveillance and control strategies in the Americas region [8].

The genetic reassortment capacity of influenza A H5 viruses provides a significant evolutionary advantage, facilitating host-range expansion and the establishment of enzootic transmission cycles. Reassortment occurs when co-infecting viral particles exchange genomic segments within a single host cell, generating progeny with novel constellations and enhanced viral fitness [9,10]. The several viral reassortment events in IAV are determined by the conformation of their segmented genome, composed of eight viral proteins: polymerase basic 2 (PB2), polymerase basic 1 (PB1), polymerase acidic (PA), HA, nucleoprotein (NP), neuraminidase (NA), M (M1 and M2) and non-structural protein (NS) (NS1 and NEP/NS2) [11]. In H5Nx viruses, this mechanism has produced new genotypes through coinfections between low- and high-pathogenicity subtypes, driving recurrent epizootics in susceptible populations. This situation is particularly alarming due to the circulation and coexistence of subtypes such as H5N1 and H5N2 in the Americas region, subtypes which have demonstrated a high adaptability to mammals and humans, generating a state of alert due to the latent possibility of generating pandemic events [12,13].

Reassortment events involving highly pathogenic H5N2 viruses were first documented in Mexican poultry outbreaks during 1994–1995. Since then, viruses of the Mexican lineage have become enzootic, causing widespread, multi-year outbreaks [14,15]. Persistent immunological pressure from long-term vaccination programs has further promoted genetic and antigenic diversification within this lineage [16]. In Mexico, the National Service for Health, Safety, and Agri-Food Quality (SENASICA), through the Mexico-United States Commission for the Prevention of Foot-and-Mouth Disease and Other Exotic Animal Diseases (CPA), has strengthened its epidemiological surveillance system in the One Health context. Given this background, the present study aims to molecularly and genetically characterize highly pathogenic H5 influenza viruses isolated from outbreaks in backyard poultry in different states in Mexico, to identify evidence of genetic reassortment between H5Nx lineages, and to evaluate their phylogenetic relationships and potential implications in the zoonotic context of the IAV.

## 2. Materials and Methods

### 2.1. Study Area

**Figure 1:**
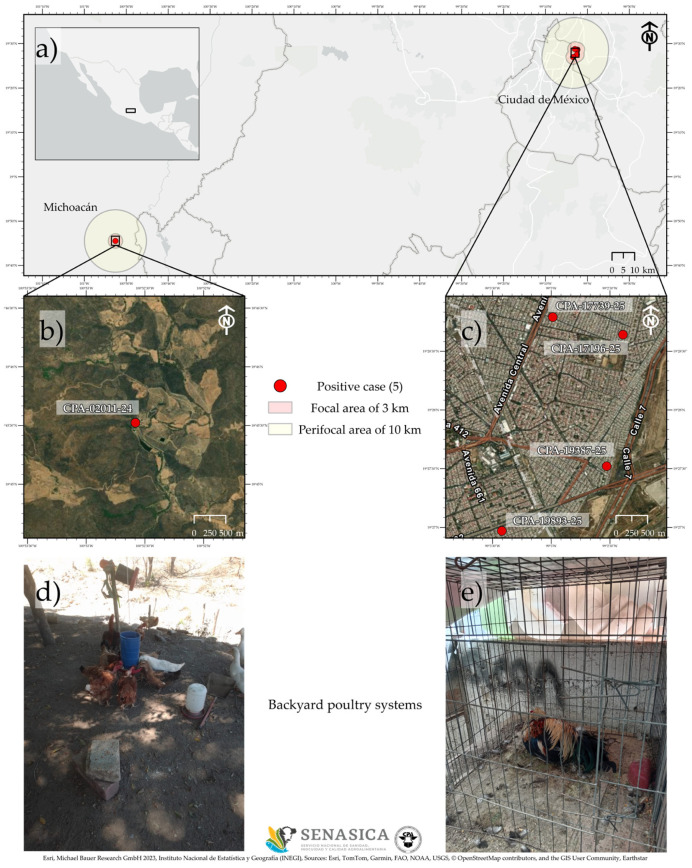
Study area of identifying highly pathogenic avian influenza A H5N2 cases in Mexico. Panel (**a**): Map showing the sampling sites with positive cases of highly pathogenic avian influenza A H5N2. Panel (**b**): Satellite imagery view of the sampling site with positive detection of highly pathogenic avian influenza A H5N2 localized in Michoacán. Panel (**c**): Satellite imagery view of three cases in the Estado de México, and one case in Ciudad de México. Panel (**d**): Picture of the site with a general characteristic of the classic backyard poultry system in Mexico, showing multiple species of domestic birds with direct interactions with the environment. Panel (**e**): Picture of the site with a general characteristic of the gamefowl backyard poultry system showing the captive conditions.

In March 2024 and September–October 2025, we sampled backyard poultry mortality notifications in three states of Mexico: Michoacán, Estado de México, and Ciudad de México (Figure 1). Backyard poultry systems are widespread across Mexico, and their characterization is challenging due to their high diversity. The primary objective of these systems is self-sufficiency, providing reliable access to animal protein and thereby contributing to food security for a significant proportion of the population. In addition, backyard poultry production may be oriented toward the breeding and maintenance of gamefowl, the sale of live birds for various zootechnical purposes, and the small-scale commercialization of eggs and meat, particularly within rural communities. The study area associated with the state of Michoacán corresponds to a rural community located in the municipality of Huetamo. The study areas in Estado de México and Ciudad de México encompass peri-urban and urban areas located in the municipalities of Nezahualcóyotl and Gustavo Adolfo Madero, respectively.

### 2.2. Sampling, Transport, and Storage

Official veterinarian service of CPA collected cloacal, oropharyngeal/tracheal swab samples, and key organs for diagnostic of avian influenza virus, which were kept chilled in PBS until they reached the official biosafety level 3 laboratory (BSL3) (Supplementary Materials). BSL3 is a High Security Isolation Unit for the Diagnosis and Research of Exotic and Emerging Animal Diseases located in Cuajimalpa de Morelos, Ciudad de México, belonging to CPA.

### 2.3. Cases and Brief Epidemiological Report

Case CPA-02011-24: On 6 March 2024, the CPA was notified of a high mortality rate among backyard poultry in the municipality of Huetamo, Michoacán (Figure 1). The population consisted of 120 birds representing three species: chickens, hens, and roosters (*Gallus gallus domesticus*), domestic ducks (*Anas* sp.), and domestic turkeys (*Meleagris gallopavo* var. *domesticus*). At the time of reporting, 117 birds across these species had died. Most deaths occurred suddenly; however, some birds exhibited clinical signs before death, including nasal discharge, diarrhea, anorexia, and prostration lasting no longer than 12 h.

Case CPA-17196-25: On 12 September 2025, a report was received from a site that raised gamefowl roosters (*Gallus gallus domesticus*) in the municipality of Nezahualcóyotl, Estado de México (Figure 1). The report included bird deaths, diarrhea, purple crests and wattles, and subsequent death. The problem started seven days before the report. Of a total of 40 birds, 27 had died, and the rest showed serious clinical signs. Necropsies were performed, identifying the main lesion as the presence of congested and hemorrhagic trachea and lungs.

Case CPA-17339-25: As part of the epidemiological follow-up to case CPA-17196-25, a gamefowl production (*Gallus gallus domesticus*) epidemiologically related to a confirmed case was inspected on 18 September 2025, in Nezahualcóyotl, Estado de México (Figure 1). The investigation was conducted because the bird owners were acquaintances who occasionally shared materials and supplies for poultry care. The owner reported the onset of clinical signs the previous day, with two mortalities and seven affected birds. Clinical manifestations included respiratory distress, ruffled feathers, prostration, and death. Necropsy revealed hemorrhagic trachea and duodenal loops.

Case CPA-19387-25: On 5 October 2025, a gamefowl production (*Gallus gallus domesticus*) was reported in Nezahualcóyotl, Estado de México (Figure 1). The owner noted two mortalities and seven clinically affected birds. Clinical signs included respiratory distress, ruffled feathers, prostration, and death; necropsy revealed tracheal and duodenal hemorrhage.

Case CPA-19893-25: On 8 October 2025, a report of mortality in gamefowl roosters (*Gallus gallus domesticus*) was attended at a property located in the Gustavo A. Madero borough, in Ciudad de México (Figure 1). The property had a total population of eight birds, all of which became sick, and one died. The birds showed clinical signs such as nasal and ocular discharge, ruffled feathers, diarrhea, and depression. Field personnel collected samples of duodenal loop, spleen, trachea, lung, and brain, as well as tracheal and cloacal swabs.

The epidemiological data of these outbreaks indicated a wide range of the mortality rate, with values of 12.5% to 97.5% and fluctuations of the lethality rate of 12.5% to 100% (Table 1). It is important to note that, as these were backyard poultry productions, the bird owners did not maintain records of productive parameters, daily mortality, bird movements, or the entry of supplies for the care and maintenance of the flocks. Nevertheless, epidemiological data were obtained through the Terrestrial Species Epidemiological Investigation Form (SIVE 02) of the National Epidemiological Surveillance System, under SENASICA. In all cases, it was recorded that no bird movements had occurred in the previous 30 days and that, due to the conditions of captivity in which the birds were kept, there was both direct and indirect contact with wild birds. For details on the procedure of sampling, as well as the main findings at necropsy, we recommend consulting the Supplementary Materials.

### 2.4. Sample Screening, Molecular Characterization, and Viral Isolation

Samples were processed as a screening by qRT-PCR following virus isolation and pathogenicity testing. For this activity, viral RNA extraction was performed using the Nucleic Acid Extraction Kit (B200-32, Zybio, Chongqing, China) and the EXM 3000 automated system (Zybio), according to the manufacturer’s instructions. Detection of the matrix (M) gene was carried out by real-time reverse transcription PCR (qRT-PCR) on an Applied Biosystems 7500 platform, using the AgPath-ID™ One-Step RT-PCR Kit (Applied Biosystems #4387391, Waltham, MA, USA) in a final reaction volume of 25 µL. The amplification conditions consisted of reverse transcription at 45 °C for 10 min, initial denaturation at 95 °C for 10 min, followed by 45 cycles of 94 °C for 1 s and 60 °C for 30 s. The diagnostic algorithm involved the detection of the M gene, HA gene (H5 and H7), NA gene, and the characterization of the highly pathogenic genotype (clade 2.3.4.4b) [17]. We recommend consulting primers and probes used for the detection of avian influenza virus genes in the Supplementary Materials.

To confirm the presence of infectious particles of IAV, viral isolation was performed by inoculating field samples into 9–11-day specific pathogen-free (SPF) embryonated eggs via the allantoic cavity. Following incubation at 37 °C for 48–72 h, the allantoic fluids were harvested, monitored for embryo mortality, and subsequently tested for subtype identification.

Viral identification was performed by hemagglutination inhibition (HI) and neuraminidase inhibition (NI) assays following standard protocols obtained from the NVLS laboratory of the USDA. The HI assay was used to determine the hemagglutinin subtype by assessing the ability of subtype-specific reference antisera to inhibit virus-induced agglutination of erythrocytes. Neuraminidase subtype determination was conducted using the NI assay, which measures the inhibition of neuraminidase enzymatic activity by specific antisera. Together, these assays were used for antigenic characterization and definitive confirmation of IAV subtypes in this study.

The IVPI was determined according to the standard procedure established by the World Organization for Animal Health [18]. Ten Six-week-old SPF chickens were intravenously inoculated with a diluted virus suspension and monitored daily for 10 days. Clinical signs were scored as follows: 0 = normal, 1 = sick, 2 = severely sick, 3 = dead. The IVPI was calculated as the mean score per bird per observation, with values >1.2 indicating the presence of HPAI viruses.

### 2.5. Sequence Analysis

Nucleic acid was extracted manually using the HighPureViral RNA kit (Roche Life Science, Basel, Switzerland) as per the manufacturer’s instructions. The sequencing of influenza A requires whole-genome amplification of the virus using two previously published primer sets, MBTuni-12 and MBTuni-13. These primers target the conserved region of the *Alphainfluenzavirus influenzae* genome for efficient and reliable amplification [15,19]. DNA libraries for next-generation sequencing were prepared using the Nextera XT DNA Sample Preparation Kit (Illumina, San Diego, CA, USA) and sequenced by generating multiplexed paired-end sequencing libraries [15].

For the assembly of the obtained short reads, in 2024, we used SPAdes, and in 2025, IRMA [20,21]. To use SPAdes, we first performed a quality analysis with FastQC, followed by cleaning and trimming of the reads with Trim-Galore [22]. After that, we aligned the reads with databases containing each of the *A. influenzae* segments’ sequences reported in the NCBI Virus database. Reads that aligned <70% of their length were discarded. Then, we executed SPAdes on the remaining reads for each segment, exploring every available k-mer, and selecting the longest contig per segment. Finally, to obtain sequencing depths, we performed new alignments of reads of each segment to the corresponding assembled contig. To increase the efficiency of the assembly workflow, we migrated to the IRMA algorithm, maintained by the CDS agency of the USA. The raw fastq files were used as input for the algorithm, which carried out the cleaning, filtering, and assembly. According to the algorithm, the latter process was performed by a series of alignments to predefined consensus sequences of influenza viruses, followed by modifications of those consensus sequences to minimize the mismatches with the reads, and producing the final contigs. We realized the sequence analysis using all available sequences deposited in GenBank. The search filter criteria included influenza A subtype H5N2 and H5N1 in some mammal and bird hosts in the North American region. The number of sequences available varied depending on the gene analyzed. Sequence alignments were created using MAFFT v7.505 and edited with MEGA 11. Mutations at key residues of specific genes were screened using FluSurver, which identifies substitutions with phenotypic or epidemiological relevance [23].

### 2.6. Phylogenetic Analysis

Phylogenetic analyses were performed to determine the genetic relationships among the H5Nx viruses characterized in this study and reference strains available in the GenBank database. Complete or near-complete nucleotide sequences corresponding to all eight genomic segments (PB2, PB1, PA, HA, NP, NA, M, and NS) of HPAI H5 viruses detected in Mexico were included in the analysis.

Multiple sequence alignments for each genomic segment were generated using MAFFT v7 under the L-INS-i strategy. The best-fit nucleotide substitution model for each dataset was determined using ModelFinder implemented in IQ-TREE v2, based on the Bayesian Information Criterion, considering among-site rate heterogeneity modeled with a discrete gamma distribution and, when supported, a proportion of invariant sites; maximum likelihood phylogenies were inferred under the selected models with 1000 ultrafast bootstrap replicates to assess branch support.

Phylogenetic trees were visualized and edited using FigTree v1.4.4 and exported as high-resolution quality format (300 DPI). The resulting topologies were compared with representative sequences from diverse viral lineages and geographic regions to assess genetic relationships and to explore potential patterns of viral introduction or dissemination. All nucleotide sequences generated in this study were deposited in the GenBank database under accession numbers PX596316–PX596355 (Supplementary Materials).

## 3. Results

### 3.1. Sample Screening, Molecular Characterization, and Viral Isolation

The samples were screened using molecular methods, and all samples with positive results were processed using traditional virological methods. Molecular diagnosis was determined as all samples tested positive for the M protein of IAV by qRT-PCR, confirming the presence of avian influenza virus. Subsequent subtyping assays showed that all samples were positive for the H5, and further molecular characterization identified the highly pathogenic genotype clade 2.3.4.4b. These results confirmed that all isolates corresponded to HPAI H5 viruses. Molecular confirmation by qRT-PCR verified the presence of avian influenza virus, and NA subtyping identified all isolates as positive for N2. Additionally, viral isolation determined hemagglutination activity, and the virus was classified as H5N2 through HI tests and NI tests, respectively. The IVPI was performed for the five isolates with the fresh allantoic fluid, obtaining values that ranged from 2.88 to 3.0, consistent with the classification of HPAI viruses (Table 1). Moreover, sequence analysis of the HA gene revealed a multibasic cleavage site motif (PLREKRRKR/GLF), a molecular marker associated with high pathogenicity in birds as defined by OFFLU.

### 3.2. Sequence Analysis

The complete genome analysis of the eight gene segments indicated that the Huetamo strain, isolated from the CPA-02011-25 case, possesses a genomic constellation comprising six gene segments derived from the highly pathogenic Eurasian H5N1 linage as follows: segment 1 PB2, segment 2 polymerase PB1 and PB1-F2 genes, segment 3 polymerase acidic protein (PA) and PA-X protein gene, segment 4 HA gene, segment 7 M2 and M1 genes, and segment 8 nuclear export protein (NEP) and NS1 genes. These genomic constellations are consistent with viruses identified in the Americas in 2022. The NP and NA gene segments aligned with those of the low-pathogenic avian influenza virus H5N2 Mexican lineage isolated in the same year. On the other hand, the viruses isolated in 2025 from Nezahualcóyotl and Gustavo A. Madero contain a genomic constellation of five gene segments derived from the highly pathogenic H5N1 virus, corresponding to genes encoding PB2, PA, HA, M2, and NEP. The PB1, NP, and NA proteins correspond to viruses isolated in 2024 in Puebla, Estado de México, and Ciudad de México, incorporating segment 2 PB1 into the genomic constellation.

The comprehensive analysis of segments 4 and 6 was critical for the study; segment 4 HA indicated that the hemagglutinin lineage was introduced between 2022 and 2023 and carried substitutions typical of the highly pathogenic H5N1 viruses introduced into North America during 2021–2022. The L131Q mutation affects an antigenic loop in the HA head, potentially altering antibody recognition. In contrast, G284E and V285M, located near the C-terminal region, may enhance structural stability and environmental persistence. The HA retains a multibasic cleavage motif characteristic of high pathogenicity, with conserved avian receptor specificity α2,3-linked sialic acids. The 2024 isolate retained most of the mutations detected in 2023 and incorporated T52A, located within the signal peptide region, which may influence HA processing and maturation. The isolate from Huetamo, Michoacán, represented the only virus detected in 2024 and reflects the consolidation of a locally adapted Mexican lineage within the clade 2.3.4.4b H5N1 viruses.

The 2025 isolates exhibited an expanded mutational pattern that included multiple substitutions within antigenic sites (L131Q, P139S, T143A, P152S) and functionally relevant positions (A226V, T204I, I351K). Two distinct outbreak hotspots were identified in 2025: the first occurred during the winter of 2024–2025, involving viruses classified as genotype D1.1; the second took place in the autumn of 2025, affecting gamefowl productions in the municipalities of Nezahualcóyotl and Gustavo A. Madero, located within Ciudad de México and its surrounding metropolitan area. The A226V substitution has been previously associated with altered receptor-binding properties in experimental models, potentially influencing host range. However, the present study did not assess functional receptor binding or mammalian adaptation, and therefore, the zoonotic implications of this substitution remain speculative. Mutations within the stem region (I351K, N465D, K514R, V526I, and M548V) contribute to protein stability and cross-species adaptability. The conservation of the multibasic cleavage site, together with the accumulation of mutations in the receptor-binding and stem regions, reflects positive selection and the reassortment event with other H5Nx lineages.

Segment 6 (neuraminidase gene) identifies isolates from Nezahualcóyotl, Huetamo, and Gustavo A. Madero, genetically related to Mexican low-pathogenic avian influenza H5N2 viruses. Since 2020, H5N2 strains exhibited moderate variation while maintaining the ancestral Mexican lineage signature. The most recent 2025 isolates in Nezahualcóyotl and Gustavo A. Madero displayed an expanded mutation pattern, including R199N, D208N, V212T, H284Y, and F466V, located near or within the catalytic pocket, potentially enhancing substrate accommodation, oseltamivir resistance, and interspecies adaptability. Altogether, the progressive accumulation of substitutions across catalytic and framework residues supports ongoing local evolution and positive selection on the NA gene, leading to the emergence of a regionally adapted clade 2.3.4.4b H5N2 genotype in central Mexico.

### 3.3. Phylogenetic Analysis

Comprehensive phylogenetic analyses were conducted for all eight genomic segments of highly pathogenic avian influenza viruses detected in backyard poultry and other avian hosts in Mexico between 2020 and 2025. The combined results reveal a complex evolutionary scenario characterized by the introduction of novel H5 lineages, local reassortment with endemic gene segments, and subsequent regional diversification.

**Figure 2:**
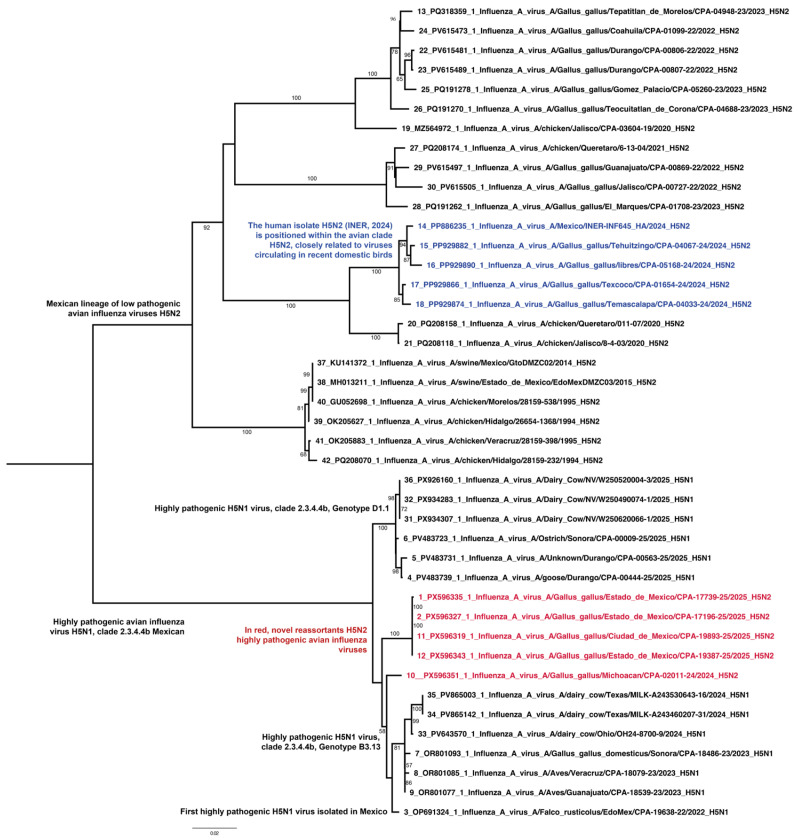
Phylogeny of the HA genes of avian-origin H5N2 viruses in central Mexico. The analysis reveals the detection of highly pathogenic H5N2 clade 2.3.4.4b in Mexico and the emergence of reassortant H5N2 viruses containing gene segments from the Mexican lineage, indicating local adaptation and genetic diversification. Sequences in red show the novel reassortant H5N2 HPAI viruses with a potential geographical structure and the sequences in blue correspond to low-pathogenic H5N2 viruses isolates in one human case and domestic birds in 2024.

Phylogenetic analysis of the HA gene demonstrated that Mexican isolates clustered within the clade 2.3.4.4b, grouping closely with highly pathogenic H5N1 viruses introduced into North America during 2021–2022. These sequences showed strong genetic similarity to HA H5 detected in domestic and wild birds in Mexico in 2022–2024 and the H5N1 viruses isolated in cattle in Texas in 2024, consistent with a recent highly pathogenic influenza A H5 transboundary introduction to Mexico (Figure 2). Importantly, Mexican H5N2 isolates did not cluster with historical Mexican H5 lineages at the HA level, confirming that the H5 gene was newly introduced rather than derived from endemic viruses.

**Figure 3:**
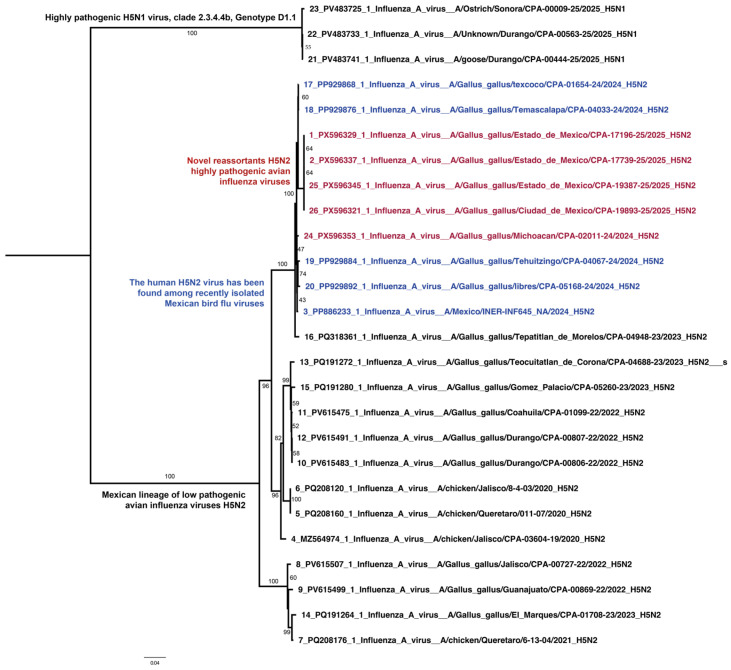
Maximum-likelihood phylogeny of NA genes from avian-origin H5N2 viruses detected in central Mexico. The NA sequences clustered within the Mexican H5N2 lineage, confirming reassortment with endemic strains. Sequences in red show the novel reassortant H5N2 HPAI viruses and the sequences in blue correspond to low-pathogenic H5N2 viruses isolates in one human case and domestic birds in 2024.

In contrast to HA, phylogenetic reconstruction of the NA gene revealed that Mexican isolates clustered with the H5N2 lineage previously circulating endemically in the country. This pattern indicates that the NA segment was locally derived, rather than co-introduced with the H5 clade 2.3.4.4b HA gene. The incongruent phylogenetic placement between HA and NA provides strong evidence for reassortment events involving endemic N2 segments and newly introduced H5 viruses. Isolates from Huetamo, Nezahualcóyotl, and Gustavo A. Madero formed a well-supported cluster, suggesting local diversification and regional adaptation of the reassortant H5N2 viruses (Figure 3).

The PB2 phylogeny showed greater genetic heterogeneity compared to other internal genes. Mexican H5N2 isolates clustered closely with contemporary H5N1 viruses detected in Mexico, including strains from domestic poultry, wild birds, and captive avian species. This topology suggests that PB2 was likely acquired through recent reassortment events, potentially reflecting selective pressures associated with host adaptation and replication efficiency. The observed diversity in PB2 contrasts with the more conserved nature of other polymerase components. The PA gene displayed the highest level of conservation among the polymerase segments. Mexican H5N2 viruses formed a tight monophyletic cluster, with several H5N1 isolates branching in proximity. This pattern supports the circulation of a stable and compatible PA lineage, facilitating reassortment of other polymerase segments without compromising replication fitness. Collectively, PB2, PB1, and PA analyses indicate that reassortment occurred within a functionally optimized polymerase complex. NP sequences clustered consistently with the endemic Mexican H5N2 lineage, showing minimal genetic divergence across geographic regions and years. This suggests long-term maintenance of a locally adapted NP segment, likely to contribute to efficient viral replication and host adaptation in gallinaceous poultry. The M gene phylogeny showed a conserved topology like NP, with Mexican H5N2 isolates forming a monophyletic group distinct from North American wild bird lineages. This pattern indicates limited recent introduction of M segments and supports the persistence of an endemic internal gene constellation. Analysis of the NS segment revealed moderate diversity but maintained clustering within Mexican H5N2 lineages. The absence of major phylogenetic incongruence with NP and M suggests that NS has co-evolved with other internal genes, contributing to immune modulation and host adaptation. The observed segment-specific phylogenetic incongruence, together with the temporal and geographic overlap of H5N1 and H5N2 viruses, supports reassortment rather than shared ancestry as the primary mechanism driving the emergence of novel H5N2 highly pathogenic avian influenza viruses in Mexico.

## 4. Discussion

The continuous emergence of novel H5 reassortant viruses reflects the extraordinary evolutionary plasticity of IAV and underscores the relevance of reassortment as a major driver of viral diversification, fitness optimization, and ecological expansion. In the Americas, the introduction of Eurasian-origin H5N1 clade 2.3.4.4b since 2021 has reshaped the regional influenza virus landscape, creating unprecedented opportunities for genomic exchange with endemic lineages [24,25]. Within this broader evolutionary framework, the present study documents the emergence and establishment of novel H5N2 reassortants in Mexico. These findings represent the first identification of highly pathogenic H5N2 reassortants containing a clade 2.3.4.4b hemagglutinin in central Mexico, providing evidence of local reassortment between recently introduced Eurasian-origin H5 viruses and endemic Mexican H5N2 lineages.

The genomic configurations identified illustrate different stages of this evolutionary mechanism of the IAV. From a theoretical standpoint, reassortment represents an adaptive strategy that allows influenza viruses to rapidly explore novel genomic constellations without the need for gradual accumulation of point mutations, an evolutionary process that occurs in different regions of the world [5,9,24,25,26,27,28,29]. The Huetamo virus, which retained a predominantly Eurasian H5N1 genetic backbone with only NP and NA derived from Mexican H5N2 viruses, likely represents an early reassortment event following the introduction of clade 2.3.4.4b into Mexico. In contrast, the 2025 viruses from Nezahualcóyotl and Gustavo A. Madero exhibited more complex reassortment patterns, incorporating PB1, NP, and NA segments from locally circulating H5N2 viruses, consistent with prolonged co-circulation and in-country viral evolution.

The acquisition of internal gene segments from endemic Mexican H5N2 viruses may confer selective advantages related to viral fitness, replication efficiency, or host compatibility. Reassortment involving PB1 is noteworthy, as this segment plays a central role in viral replication and has been frequently implicated in host adaptation and altered virulence in multiple influenza systems. Although functional assays were not conducted in this study, the repeated incorporation of PB1, NP, and NA from Mexican lineages suggests positive selection for locally adapted internal gene cassettes that may enhance viral persistence in backyard poultry environments.

Mutational analyses of the HA genes further support ongoing adaptive processes. The conservation of the multibasic cleavage site in HA across all isolates is consistent with their highly pathogenic phenotype in chickens, as confirmed by IVPI values. HA is a surface glycoprotein that mediates receptor binding to susceptible host cells and subsequent membrane fusion of influenza A viruses. Consequently, amino acid substitutions within this region of the viral genome may alter the capacity of H5 viruses to infect cells of novel hosts under specific circumstances. Substitutions at positions Q226L, V135M, N224K, T160A, P136S, A156T, T187P, and M227L have been associated with changes in host specificity [30,31,32,33]. For example, experimental evidence indicates that the Q226L substitution in the HA of highly pathogenic H5N1 clade 2.3.4.4b viruses is sufficient to shift receptor preference entirely from avian-type to human-type receptors [32]. However, the presence of such substitutions alone is generally insufficient to account for cross-species transmission of avian-adapted viruses, given the complex biological conditions, ecological interactions, and evolutionary forces that drive these events.

At the same time, the accumulation of substitutions within antigenic sites, receptor-binding regions, and the HA stem indicates sustained selective pressure, likely driven by host immunity, ecological constraints, and viral transmission dynamics. Substitutions such as A226V and T204I, while not sufficient on their own to indicate mammalian adaptation, warrant attention due to their reported associations with altered receptor binding or fusion properties in other influenza contexts [34]. Importantly, no definitive molecular markers of mammalian adaptation were detected, supporting the interpretation that these viruses remain primarily avian-adapted.

The neuraminidase gene provides additional insight into the evolutionary trajectory of these reassortants. The clustering of NA segments within the Mexican H5N2 lineage, coupled with the progressive accumulation of mutations near the catalytic and framework regions, suggests localized adaptation rather than recent reintroduction. While some substitutions identified have been associated elsewhere with altered enzymatic activity or antiviral susceptibility, their phenotypic impact in these viruses remains to be experimentally validated. Nonetheless, the persistence of a Mexican-derived N2 in combination with a Eurasian H5 HA highlights the role of endemic lineages as genetic reservoirs that can shape the evolution of newly introduced highly pathogenic viruses.

Based on the phylogenetic evidence and the information obtained during the epidemiological investigation and the genetic characterization of the isolated viruses, we hypothesize that the H5N1 and H5N2 viruses infecting poultry in Michoacán, Estado de México, and Ciudad de México originated from infected wild birds, which interact directly and indirectly with backyard poultry systems. In Mexico, these poultry systems, characterized by limited biosafety, frequent animal movements, and close contact with wild birds and humans, constitute ideal interfaces for viral maintenance, reassortment, and spillover. In the absence of protective antibodies against the H5Nx, such interactions lead to outbreaks with high mortality and lethality, thereby promoting viral adaptation and evolution. The establishment of reassortant H5N2 viruses in these settings raises concerns not only for poultry health but also for broader animal-human interfaces, reinforcing the importance of One Health approach-oriented surveillance strategies already implemented for IAV in Mexico [13,35,36,37]. The first confirmed human infection with an influenza A H5N2 virus worldwide was reported in Mexico in 2024, and this case provides an important eco-epidemiological find, as genomic analyses revealed a close evolutionary relationship between the virus detected in the human patient and contemporaneous H5N2 viruses circulating in poultry in the Central Mexico region [13]. Although sustained human-to-human transmission has not been documented and the overall public health risk remains low, this zoonotic event highlights the importance of the novel reassortant H5N2 HPAI viruses at the domestic animals–humans–wild birds interface.

A clear gap exists in molecular surveillance and in the ecological understanding of IAV in wild birds in Mexico, emphasizing the need to investigate the roles of species belonging to understudied taxonomic orders with high interaction with backyard poultry systems. Wild bird species at greatest risk of H5N1 infection are non-migratory, gregarious, habitat generalists, tolerant of anthropogenic disturbances, and occupy higher trophic levels due to their complex roles in ecological interaction networks [38]. It is therefore critical to highlight the exposure risk of specific understudied bird orders beyond Anseriformes, including Accipitriformes, Ciconiiformes, Passeriformes, and Pelecaniformes [39]. From an eco-epidemiological perspective, the detection of these reassortant viruses in backyard poultry systems reveals a high-risk scenario for the persistence and transmission of IAV, underscoring the urgent need to expand official active surveillance across diverse wild bird species and the backyard poultry distributed throughout Mexico.

In the Mexican context, these findings expand upon previous knowledge derived from the H5N2 highly pathogenic outbreaks of the 1990s and subsequent enzootic circulation under vaccination pressure. Unlike earlier Mexican H5N2 viruses, the reassortants described here combine a contemporary Eurasian H5 HA with locally adapted internal genes, creating novel genomic constellations with uncertain epidemiological behavior. This study therefore provides the first molecular evidence of clade 2.3.4.4b H5N2 reassortants in Mexico, demonstrating that local evolutionary processes are actively shaping the genetic landscape of H5 viruses in the country.

## 5. Conclusions

This study reports the emergence of novel H5N2 reassortant viruses in central Mexico, resulting from interactions between highly pathogenic H5N1 clade 2.3.4.4b and endemic low-pathogenic H5N2 lineages. The distinct genomic constellations identified, ranging from early-stage reassortants to more complex combinations involving PB1, NP, and NA, demonstrate active viral exchange within backyard poultry systems. These findings emphasize the critical role of informal production environments as ecological niches that facilitate reassortment and sustain viral diversity. Mutational patterns in HA and NA further reveal ongoing adaptation and selective pressures consistent with extended regional circulation. The emergence of these reassortants underscores the urgent need to strengthen genomic surveillance programs, particularly in regions where influenza lineages overlap and biosafety measures are limited. Continuous monitoring will be essential to assess the evolutionary trajectory, pathogenic potential, and epidemiological impact of these viruses, as well as their implications for poultry health, zoonotic transmission, and pandemic risk.

## Figures and Tables

**Table 1:** General information and epidemiological data of positive cases of highly pathogenic avian influenza A H5N2 in Mexico.

Case	Date of Sample Collection	Backyard Poultry System	Location in the Dwelling	Backyard Poultry Species	Municipality	State	Influenza A Vaccination	Potential Source of Infection	Signs	Intravenous Pathogenicity Index (IVPI)	Genotype	Population	Sick Animals	Dead Animals	Mortality Rate	Lethality Rate
CPA-02011-24	6 March 2024	Self-sufficiency	Yard	*Anas* sp., *Gallus gallus domesticus,* and *Meleagris gallopavo* var. *domesticus*	Huetamo	Michoacán	Absent	Wild birds	Neurological, respiratory, and gastrointestinal	2.88	H5N2	120	117	117	97.5%	100%
CPA-17196-25	12 September 2025	Gamefowl	Dwelling rooftop	*Gallus gallus domesticus*	Nezahualcóyotl	Estado de México	Absent	Wild birds	Respiratory and gastrointestinal	3	H5N2	40	29	27	67.5%	93.1%
CPA-17739-25	18 September 2025	Gamefowl	Dwelling rooftop	*Gallus gallus domesticus*	Nezahualcóyotl	Estado de México	Absent	Wild birds	Respiratory	3	H5N2	9	9	2	22.2%	22.2%
CPA-19387-25	5 October 2025	Gamefowl	Dwelling rooftop	*Gallus gallus domesticus*	Nezahualcóyotl	Estado de México	Absent	Wild birds	Respiratory	3	H5N2	7	7	2	28.6%	28.6%
CPA-19893-25	8 October 2025	Gamefowl	Dwelling rooftop	*Gallus gallus domesticus*	Gustavo A. Madero	Ciudad de México	Absent	Wild birds	Respiratory and gastrointestinal	3	H5N2	8	8	1	12.5%	12.5%

## Data Availability

All genome sequences generated in this study have been deposited in GenBank under accession numbers PX596316–PX596355. Additional data supporting the findings of this work are available from the corresponding author upon reasonable request.

## Supplementary Materials

The following supporting information can be downloaded at: https://www.mdpi.com/article/10.3390/v18030337/s1, Figure S1: Sampling and media for preservation and transport of sample; Figure S2: Biosafety measures for the sampling in accordance with international guidelines for avian influenza field investigations; Figure S3: Necropsies and pathological findings in the avian influenza field investigations; Figure S4: Phylogenetic relationships of the PB2 segment; Figure S5: Phylogenetic analysis of the PB1 gene (segment 2); Figure S6: Phylogenetic relationships of the PA gene; Figure S7: Maximum-likelihood phylogeny of the NP gene; Figure S8: Phylogenetic tree of the matrix gene; Figure S9: Maximum-likelihood phylogeny of the NS gene; Table S1: Highly pathogenic avian influenza (H5N2) was included in the study, along with its associated GenBank entries; Table S2: Primers and probes are used for the detection of avian influenza virus genes. Sampling and media for preservation and transport of samples. List of primers and probes used for the detection of avian influenza virus genes. Phylogenetic trees of all segments of the isolated viruses, except for HA and NA, are presented within the content of the article.
