# Supplementary material for: Novel Reassortant H5N2 Highly Pathogenic Avian Influenza Viruses from Backyard Poultry in Mexico
Source: Viruses. 2026 Mar 10;18(3):337. doi: 10.3390/v18030337 (PMC13030139; doi:10.3390/v18030337)
Supplement: Supplementary file 1 [file viruses-18-00337-s001.zip › viruses-4081209-supplementary.pdf]

Supplementary materials.

Tables.

Table S1. Highly pathogenic avian influenza (H5N2) was included in the study, along with its associated GenBank entries.

State	Municipality	Year	Description sequence in GenBank	GenBank ID
Michoacán	Huetamo	2024	Influenza A virus (A/Gallus gallus/Michoacan/ CPA-02011-24/2024(H5N2))	PX596348 - PX596355
Estado de México	Nezahualcóyotl	2025	Influenza A virus (A/Gallus gallus/Estado de Mexico/CPA-17196-25/2025(H5N2))	PX596324 - PX596331
Estado de México	Nezahualcóyotl	2025	Influenza A virus (A/Gallus gallus/Estado de Mexico/CPA-17739-25/2025(H5N2))	PX596332 - PX596339
Estado de México	Nezahualcóyotl	2025	Influenza A virus (A/Gallus gallus/Estado de Mexico/CPA-19387-25/2025(H5N2))	PX596340 - PX596347
Ciudad de México	Gustavo Adolfo Madero	2025	Influenza A virus (A/Gallus gallus/Ciudad de Mexico/CPA-19893-25/2025(H5N2))	PX596316 - PX596323

Table S2. Primers and probes are used for the detection of avian influenza virus genes.

Viral protein	Primer
M	M-124 RV: 5′-TGC AAA AAC ATC TTC AAG TCT CTG-3′
M	M+25 FW: 5′-AGA TGA GTC TTC TAA CCG AGG TCG-3′
M	M+64 Probe: 5′-FAM–TCA GGC CCC CTC AAA GCC GA–BHQ-3′
H5 HA	RV-H5: 5′-AGA CCA GCT ACC ATG ATT GC-3′
H5 HA	H5+1456 FW NA: 5′-ACG TAT GAC TAT CCA CCA TAC TCA-3′
H5 HA	H5+1456 FW EA: 5′-ACG TAT GAC TAC CCG CAG TAT TCA-3′
H5 HA	H5+1592 FW MEX: 5′-AAT CAA CAG GGA CTT ATC AGA TAC-3′
H5 HA	Probe 1637: 5′-FAM–TCA ACA GTG GCG AGT TCC CTA GCA–BHQ1-3′
HP H5	H5-R2: 5′-ATC AAC CAT TCC CTG CCA-3′
HP H5	H5-F3: 5′-CCT TGC GAC TGG GCT CAG-3′
HP H5	Probe: 5′-FAM–AGA AGA AAR AGA GGG CTG TTT GGG GCT–BHQ1-3′

Figures.

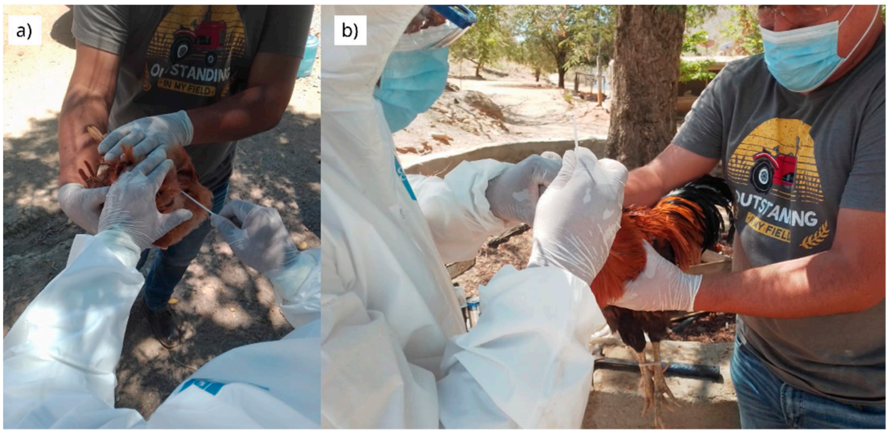

Figure S1. The production units where increased bird mortality was reported were visited as part of the passive epidemiological investigation. Upon arrival, an interview was conducted with the bird owner to collect relevant epidemiological information, including the onset of clinical signs, mortality patterns, bird movements, and recent biosafety practices. Sampling site in Huetamo, Michoacán. a) Cloacal swab collection. b) Oropharyngeal/tracheal swab collection.

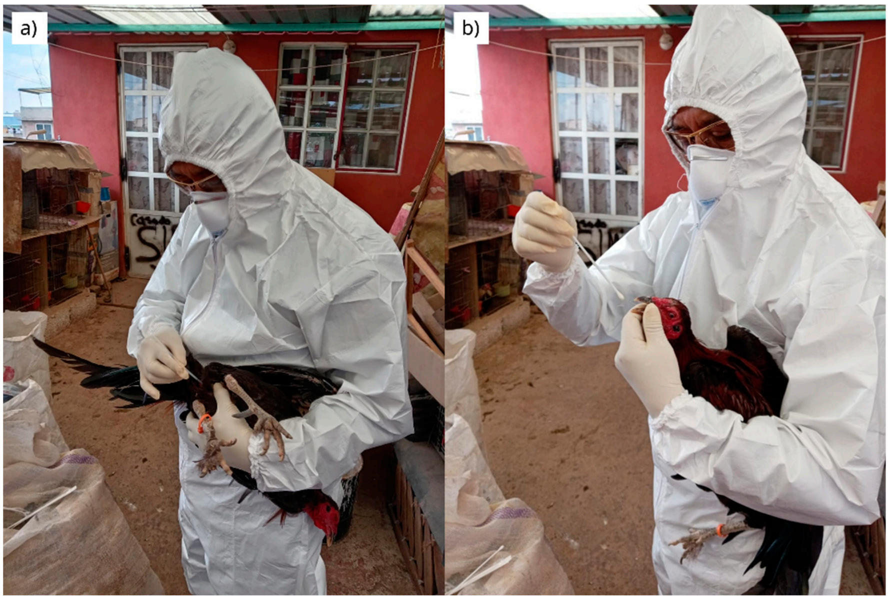

Figure S2. Strict biosafety measures were implemented before entering the premises, in accordance with international guidelines for avian influenza field investigations. Personnel used full personal protective equipment (PPE), including disposable Tyvek® hooded coveralls, face masks, protective goggles, rubber boots, plastic boot covers, and nitrile gloves, to prevent both zoonotic exposure and

mechanical spread of the virus between production units. Sampling site in Gustavo Adolfo Madero, Ciudad de México. a) Cloacal swab collection. b) Oropharyngeal/tracheal swab collection.

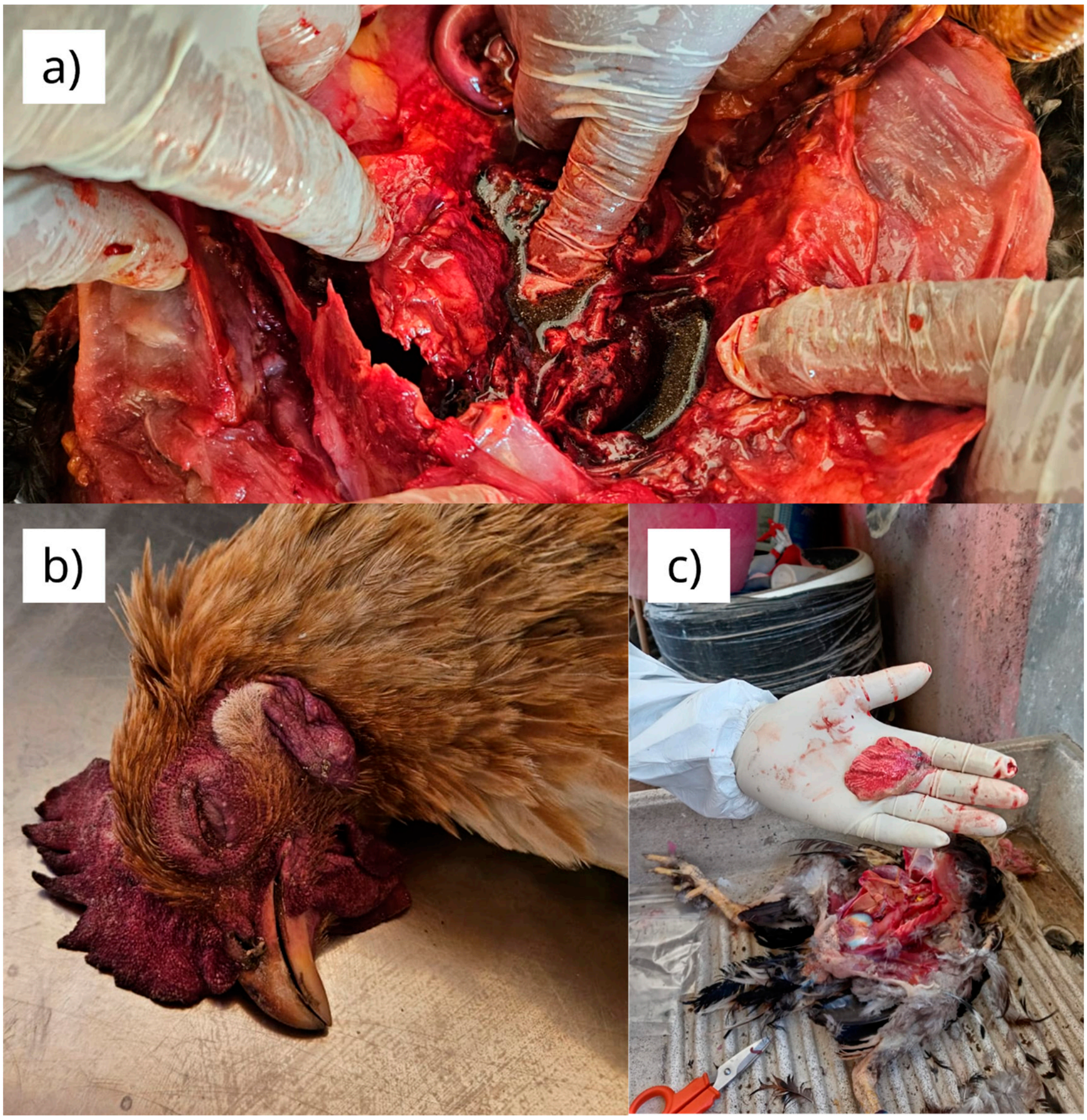

Figure S3. Bird carcasses were subjected to a systematic clinical examination to identify gross lesions consistent with avian influenza infection, such as subcutaneous hemorrhages in the head, comb, wattles, and hock joints. Necropsies were performed under biosafety conditions, and representative organ samples—including trachea, lung, spleen, and duodenal loops—were collected. These tissues were selected to ensure sampling from both respiratory and systemic sites, which is critical for detecting highly pathogenic avian influenza viruses. Samples were placed in pre-labeled, air-tight bags to preserve sample integrity and traceability. In each case, several investigations were assigned for diagnosis

in the laboratory. This figure shows a pathological postmortem outcome in the cases in the Estado de México and Ciudad de México.

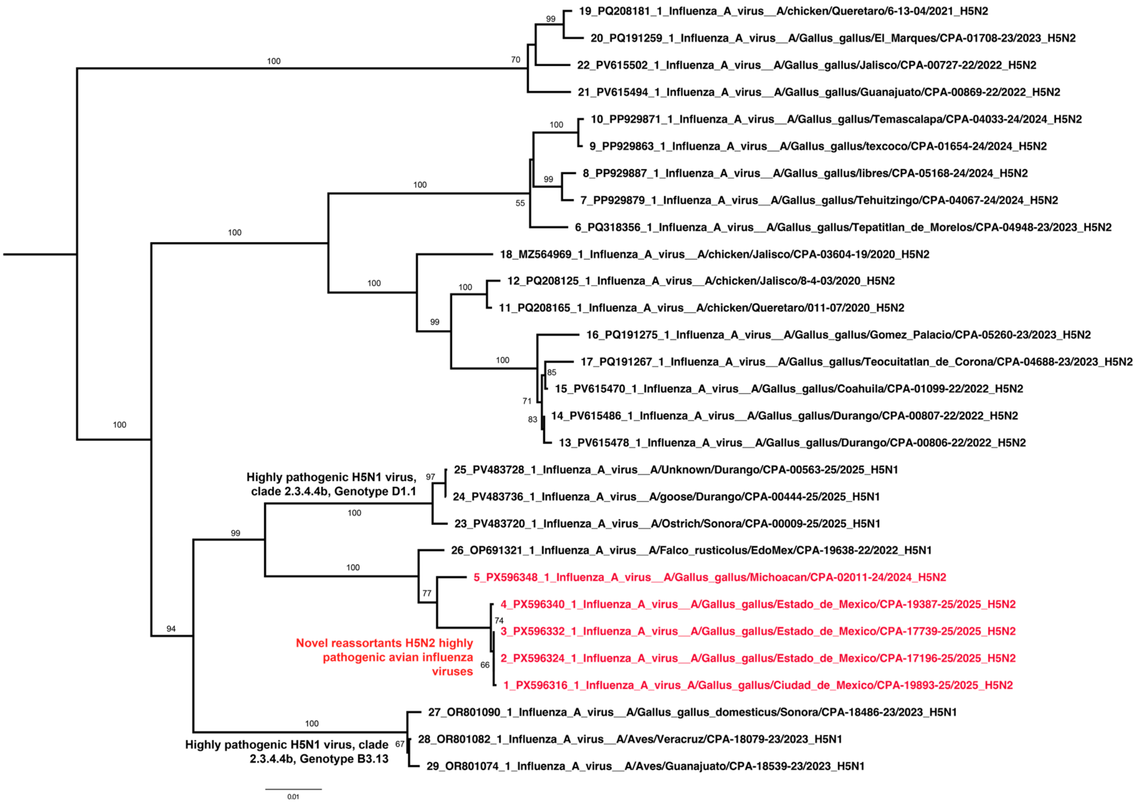

Figure S4. Phylogenetic relationships of the PB2 segment reveal reassortment between H5N2 and H5N1 highly pathogenic avian influenza viruses in Mexico.

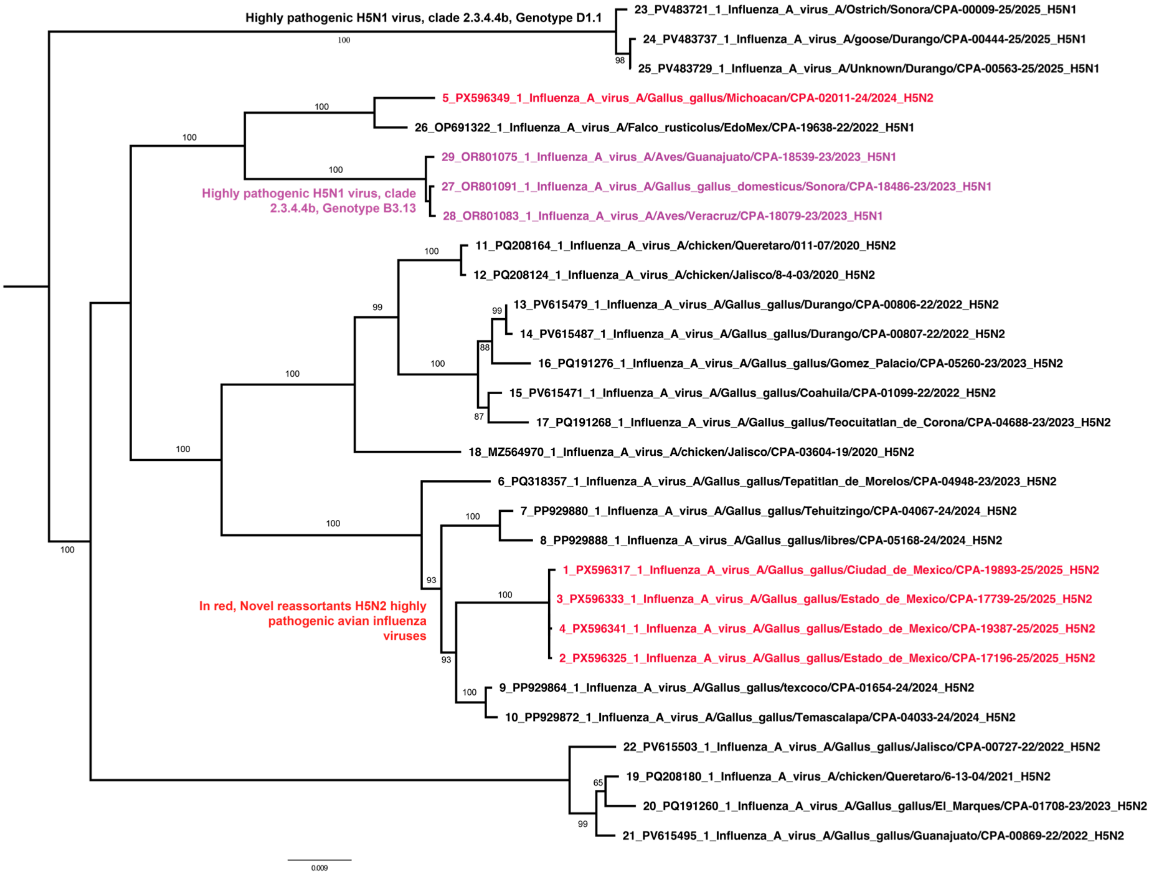

Figure S5. Phylogenetic analysis of the PB1 gene (segment 2) revealed a well-supported clade of H5N2 highly pathogenic avian influenza viruses closely related to contemporary H5N1 strains, indicating the circulation of a conserved polymerase backbone and supporting reassortment events in backyard poultry in Mexico.

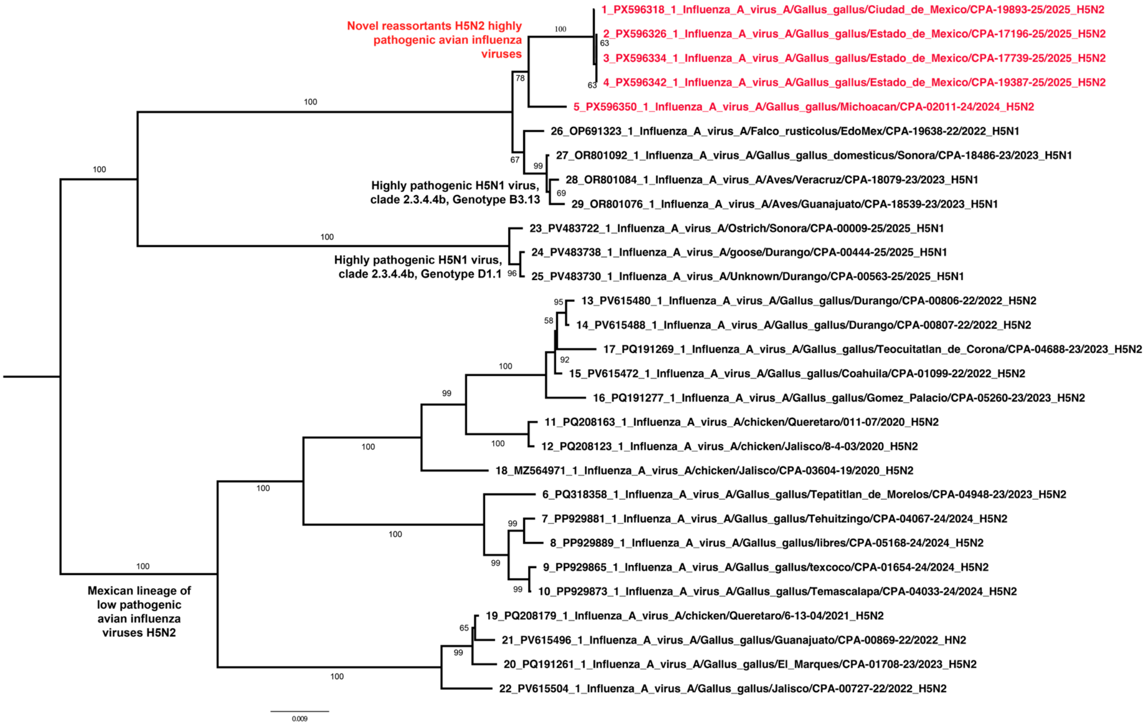

Figure S6. Phylogenetic relationships of the PA gene among Mexican H5N2 and closely related H5N1 avian influenza viruses.

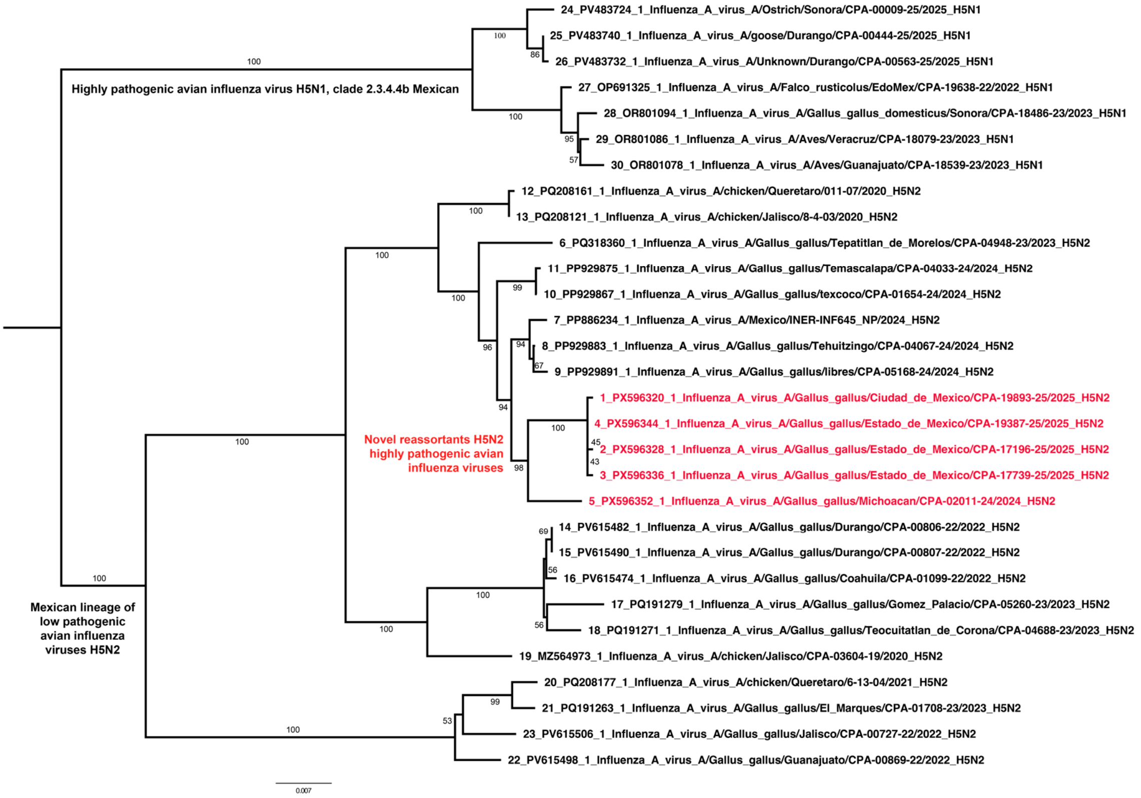

Figure S7. Maximum-likelihood phylogeny of the NP gene of H5N1 and H5N2 highly pathogenic avian influenza viruses from Mexico.

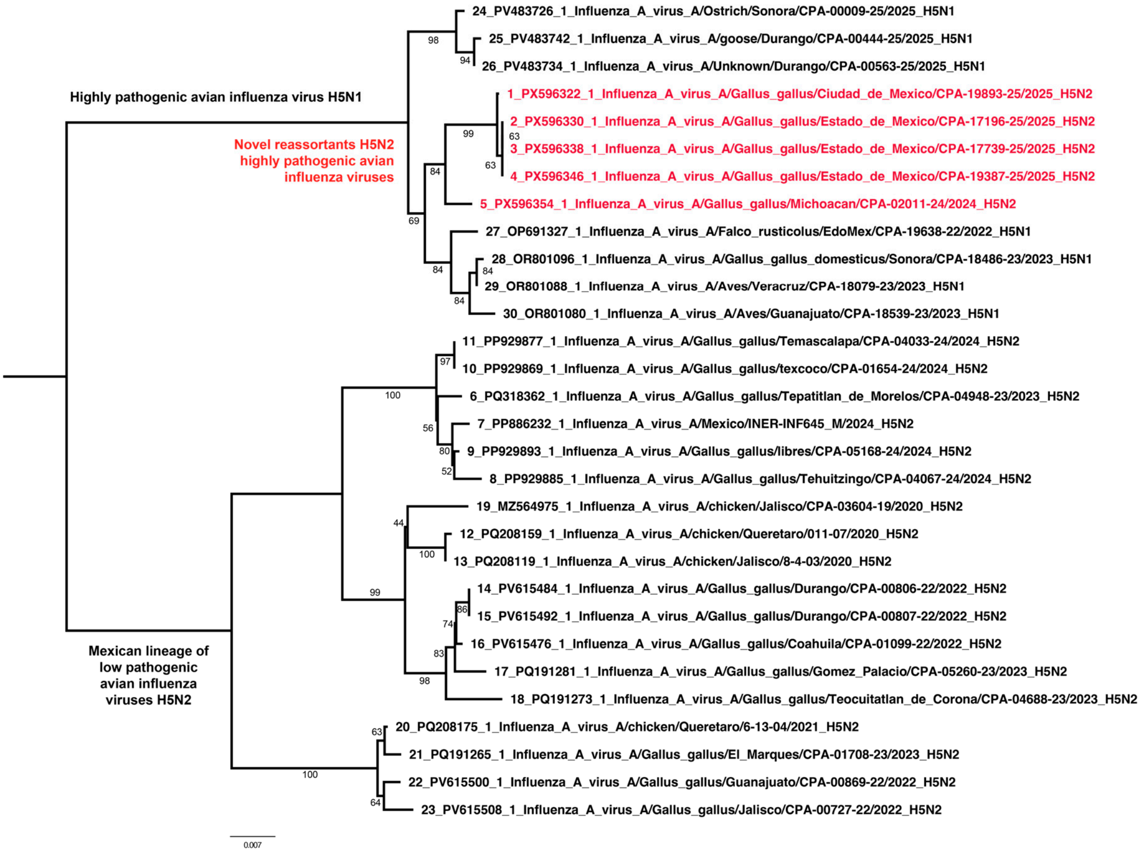

Figure S8. Phylogenetic analysis of the matrix gene of highly pathogenic H5 avian influenza viruses from Mexico.

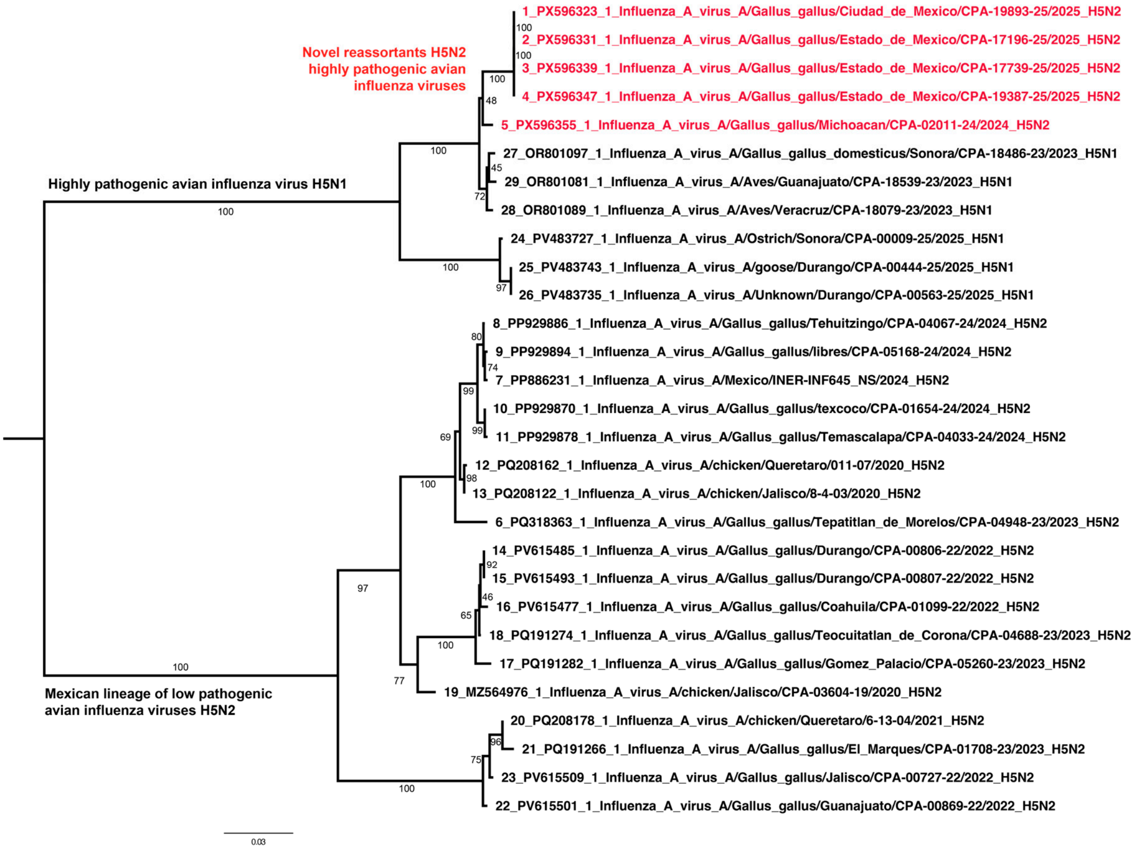

Figure S9. Maximum-likelihood phylogeny of the NS gene of H5N1 and H5N2 highly pathogenic avian influenza viruses from Mexico.
